# A systematic review about the performance indicators related to ball possession

**DOI:** 10.1371/journal.pone.0265540

**Published:** 2022-03-17

**Authors:** Si hang Wang, Yang Qin, You Jia, Kwetche Emmauel Igor

**Affiliations:** Department of Physical Education, Tianjin University of Sport, Tianjin, China; Queen Mary University of London, UNITED KINGDOM

## Abstract

The purpose of this review was to investigate the influence of the performance indicators related to ball possession on the match outcome and team performance. Following the PRISMA 2020 (Preferred Reporting Items for Systematic Reviews and Meta-analyses) guidelines, this systematic review searched for literature on Web of Science, Scopus and Pub Med database, the publication date of the retrieved literature is set from January 2000 to December 2020. The keywords ‘football’ or ‘soccer’ were each paired with the following terms: ‘ball possession’, ‘ball recovery’, ‘ball regain’, ‘transition’, ‘playing style’, ‘possession zone’, ‘duration’ and ‘running performance’. The search returned 2,436 articles. After screening the records against set criteria, 75 analysis were made, and their technical and physical indicators were identified. Through analysing ball-possession related variables, the review concluded that the match outcome is not related to ball possession percentage. The ball possession percentage is not dominant to predict the match success. The status of ball possession percentage can affect the team’s performance in passing, organizational and running distance with the ball possession. There are league differences in ball possession strategies and duration. The frequency and offensive efficiency of direct ball recovery types are higher than indirect types. Ball possessions regained in the defensive third were higher than the final third. However, there remain some limitations such as the difference in the definition of concepts and sample participants, only a few studies consider the influence of situational variables and lack of in-depth analysis on ball possession strategy. Therefore, further study should adopt a more comprehensive approach, establishing a new connection between possession strategy and more technical and tactical indicators.

## Introduction

In order to better understand the constraints of promoting team success in football, performance analysis plays a very important role in team sports [1]. Match performance is the result of dynamic interactions of physical, technical and tactical actions and movements from all competing players [2]. Match success is achieved by the combination of coaches’ teaching philosophy and the technical and tactical performance of players during the competition [3]. Although team success is complex and multifactorial, technical indicators have been found to predict team success more accurately than physical indicators [4]. More specifically, ball possession, number of shots, shots on target, number of passes and pass completion rates are all associated with team success [5].

Ball possession was regarded as a popular performance indicator in a football match [6]. Several studies have revealed that ball possession has positive effects for a team to achieve match success [7–11]. Teams with more ball possessions mean that they can organize more attacks and create more opportunities for goal scoring. In addition, the teams that had long possession time can firmly grasp the initiative of the match, exert greater psychological and physiological load on opponents, and thus improve the chances of match success [12]. However, scoring goals is the ultimate variable that determines the match outcome, the number of goals was determined by the shooting quality rather than ball possessions [13]. Moreover, football is a sport with a low frequency of goal scoring, and it only accounted for 1% of ball possessions in elite matches [14]. Therefore, in order to make full use of the 1% of ball possessions, it is very important to analyze the characteristics of ball possession, especially in successful attacks.

In this context, in the past decade, many studies showed that the ball possession percentage of the successful teams was higher than the unsuccessful teams [4,15,16]. For example, during the 2010 World Cup tournament, the national team of Spain won the championship with the highest average possession percentage of 66.3%, while the German national team won the championship with the highest average possession rate of 56.7% in the 2014 World Cup [8]. However, in recent years, the role of possession percentage in the analysis of technical indicators has gradually weakened. Many studies have pointed out that possession rate does not reflect the real situation of the game, the number and percentage of ball possessions do not mean more opportunities for shooting and scoring [17]. In the 2018 World Cup, the France national team won the World Cup with less than 50% possession percentage per match. Therefore, the value and role of ball possession need in-depth study.

At present, increasing studies try to establish the relationship between the characteristics of ball possession and team performance [18,19]. Some studies through the relationship between ball possession and technical [5] (i,e., passing, shooting, aiming and scoring) and physical indicators [20,21] (i,e., total running distance, high-intensity running distance, running distance with and without balls) to explore its value and function. In addition, the ball possession strategy is reflected by the characteristics of the offensive organization per possession [22,23]. Previous studies divided the playing styles into direct attack, counterattack and elaborate attack [24–26]. The duration of ball possession represents the complexity of the team’s attack, and it is also an important variable to evaluate the features of ball control [27–29]. However, the ball possession will not be obtained in vain, many studies attached great importance to the ball recovery patterns [30–32]. The research on the type and area of ball recovery will benefit the teams to regain the ball possession more efficiently when defense organization, and handle it more cautiously and reasonably when attacking, so as to improve the offense efficiency of the teams.

However, there are many studies about the indicators related to ball possession, and many contributions have been made, while there was not exist the systematic review on ball possession. Therefore, the aim of this paper is to systematic review the impact of performance indicators related to ball possession on football matches, investigate the relationship between ball possession related indicators and match outcome and team performance, clarify the role of ball possession in performance analysis, and summarize the achievements and limitations that researchers have made. Additionally, coaches could utilize these information to establish trends and objectives for teams and players in training and competition in order to enhance team performance.

## Method

### Design

The present systematic review of the studies related to ball possession was executed according to the PRISMA (Preferred Reporting Items for Systematic reviews and Meta-analyses) statement [33]. The publication date of the retrieved literature is set from January 2000 to December 2020. In order to improve the efficiency and accuracy of the search, ensure the quality of the articles. The databases of Web of Science, Scopus and Pub Med were searched by using the keywords ‘football or soccer’ and combining the following terms, such as ‘ball possession’ ‘ball recovery’, ‘ball regain’, ‘transition’, ‘playing style’, ‘possession zone’, ‘possession duration’ and ‘running performance’. Each of these keywords was first carried out independently and then combined into Boolean search using the AND operator. In order to ensure maximum retrieval of articles, the keywords in all fields were searched and extracted the needed information for this study.

### Inclusion and exclusion criteria

The following inclusion criteria for these articles were used: (1) the research variables were performance indicators related to ball possession; (2) the population was limited to healthy professional male or female adult football players and (3) the language of the article was English. The articles were excluded if they: (1) the sample were children or adolescents (under 18 years); (2) the research has no data support (3) were conference abstracts; and (4) the article was written in other language and did not provide an English abstract and method information. If there was a disagreement on the inclusion of articles between the two independent reviewers, the final decision was delivered to the senior author due to the greater experience on these matters. In the process of screening articles, the assessment of eligibility of the articles was performed by one review author. All articles were screened from titles and abstracts. Once there is ambiguity or indecision, two other reviewers will be invited to judge the disagreement, and the differences between inclusion or exclusion of research will be resolved through consensus.

### Quality of the articles

As in previous research [26], to make a fair comparison between studies of different designs, the decision was taken to calculate a percentage score as a final measure of methodological quality. In the present study, the quality score of all included articles was evaluated by two authors from the following 13 critical components: (1) clarify the purpose of the research; (2) relevant literature review; (3) rationality of research design; (4) participants; (5) rationality of sample size; (6) informed consent; (7) reliability and validity of measurement results; (8) detailed description of experimental method; (9) research results; (10) analysis of research methods; (11) theoretical connection; (12) conclusion; (13) implication. Then sum up the binary scores of each item, calculate the final average score, and present it as a percentage to reflect the quality standard of the article. The standard classification of the score is as follows: low quality of research method scored ≤ 50%; good quality of research method scored between 51% and 75%; and high quality of research method scored ≥ 75%. The scoring and classification methods used in this paper are consistent with the statistical methods used in previous literature reviews. By calculating the Kappa value of Cohen, an independent reliability analysis between raters was made for the quality scores [34].

### Data extraction

From each study, relevant data were extracted by one review author and checked by a second author. Disagreements between the two authors were resolved by discussion and if no agreement could be reached, a third author would make the final decision. The following information was extracted from each included study: (1) the study sample, i,e., the season and location of the leagues, the number of players and matches; (2) the purpose of the study; (3) ball possession related variable analysed, i,e., ball possession percentage, possession time, ball recoveries; (4) main results, the impact of ball possession related indicators to the team performance.

## Results

### Search results

**Fig 1:**
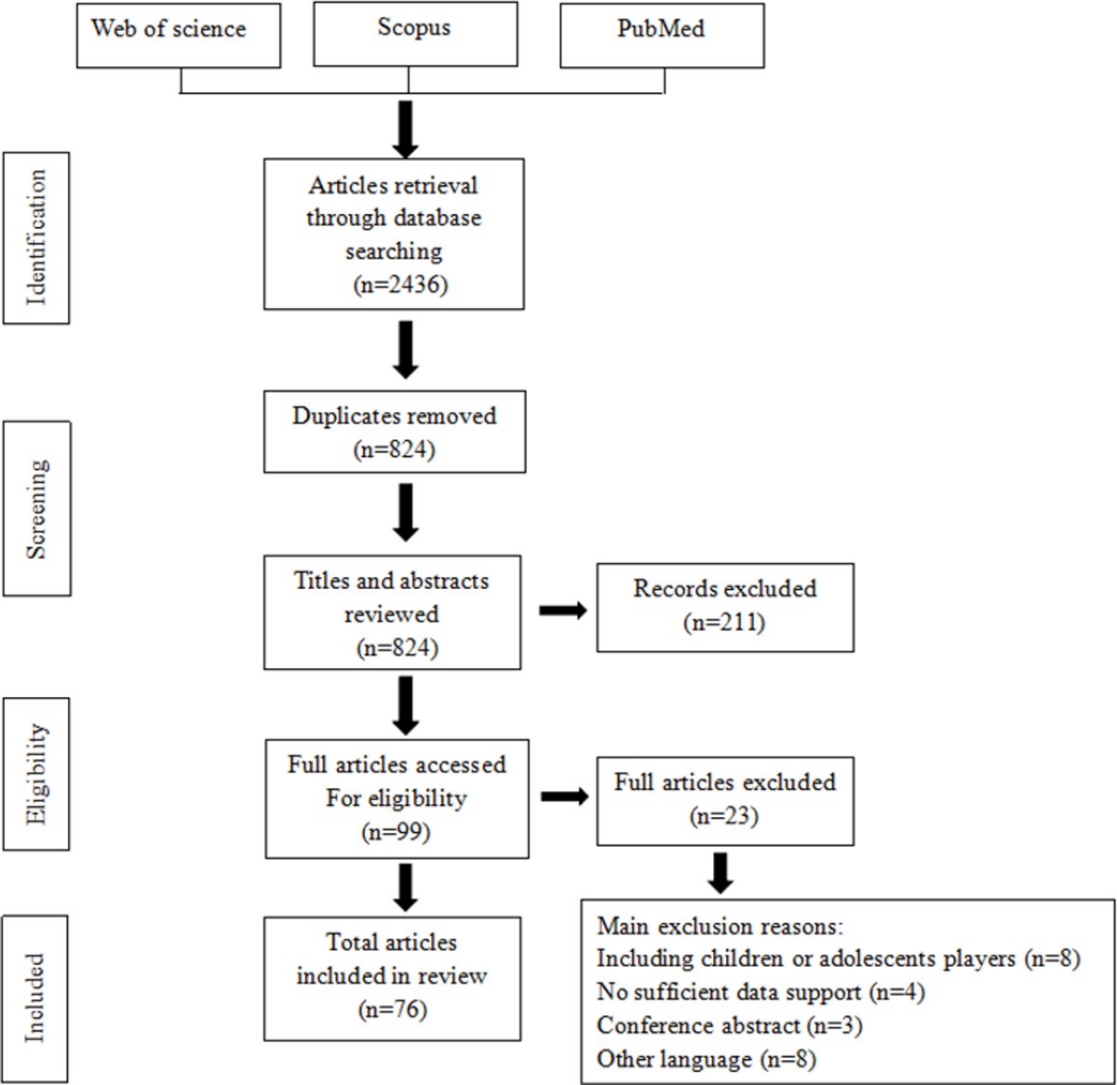
Flow diagram of study selection process.

By searching keywords on the Web of Science, Scopus and Pub Med, 2,436 articles were initially searched, and then 824 duplicate articles were eliminated. Then the articles were screened out based on screening the titles and abstracts. After excluding the studies with small sample size, the sample participants were young or amateur football players, and the scale of matches were small-sided games, only 71 articles were left. Subsequently, after manually searching related journals and reference pages, it was found that 5 articles were of good quality and met the selection criteria, but they were not included in the review list. Eventually, after adding these 5 articles, a total of 76 articles were comprehensively reviewed. The process of screening the primary documents (see Fig 1) is shown in the following PRISMA flow diagram [35].

### Quality of the articles

In a previous study, Sarmento et al. [36] proved the quality of the literature through the database of Web of Science. In the present review, according to the scores of 76 articles given by two authors, it was concluded that the average quality score of all articles is 85.9%. In previous studies, there has never been a research method that can achieve the highest score of 100% or less than 50%. 5 studies were classified as having good methodological quality (the quality score is between 51% and 75%), while 71 studies had excellent methodological quality (the quality score is greater than 75%). The inter-rater reliability analysis obtained a Kappa value of 0.88, which indicated that the consistency among the observers was very good [26]. The main defects of methodological quality involve rationality of research sample size and confirmation and description of research limitations.

### Data organization

All included studies were published ranged from 2002 to 2020, 19 articles (25%) were published from 2002 to 2012, while 57 articles (75%) were published from 2013 to 2020. The geographic origins of the included studies were: Spain(n = 22), England (n = 21), Germany (n = 7), France (n = 3), Italy (n = 3) and other countries (n = 10). In addition, 25 studies analysed World Cup matches, while 16 studies analysed UEFA Champion League matches, 6 studies analysed European Football Championship matches, comprising teams from different European countries and clubs. The sample participants of this review are all professional football players. The participants of most studies (n = 73) were male football players, only 4 studies involved female football players. Since the majority of studies only record the number of matches, but neglect the number of players, this review can not calculate the number of participants, and the number of matches observed varied from 6 [37] to 6,078 [38]. The variables related to ball possession were: ball possession (n = 21), ball possession strategies (n = 17), ball possession duration (n = 9), ball recovery patterns (n = 15), running performance (n = 14). The types of reviewed studies included: descriptive analysis (n = 17), comparative analysis (n = 29) and predictive analysis (n = 30).

### Research topic

#### Percentage of ball possession

Ball possession is the ratio of the possession time of one team to the total possession time of two teams, and it is one of the factors that is used to examine which team holds the initiative and the rhythm of the match [38]. Generally, the successful teams are usually able to get higher ball possession percentage. but the ball possession percentage is only one of the technical variables, and it needs to be combined with other variables for comprehensive analysis.

#### Ball possession strategy

The ball possession strategy was defined as the style of play at these five moments during the match [39], such as established attack, transition from defense to attack, transition from attack to defense, established defense and set play.

#### Ball possession duration

The duration of ball possession is the epitome of the team’s offensive tactics, and the playing style of the team is composed of the offense or defense characteristics per possession. Generally, the duration of ball possession is divided into three categories, that is, 0–5 seconds, 5–12 seconds and >12 seconds [9,19,40–42]. Investigating the characteristics of ball possession time is conducive to understanding the offensive patterns of modern football.

#### Patterns of ball recovery

Ball recovery was regarded as one of the reliance for successful teams by sports scientists, that is, on the type of ball recovery and the area where it occurs [30]. Ball recovery is particularly important because it symbolizes the end of the defensive phase and the initiation of the offensive stage. If a team cannot regain the ball possession, which means the team has no chance of goal scoring. A successful chance to regain the ball possession not only depends on the excellent performance of the defender but also on the offensive performance and efficiency of the attacking players. Therefore, it is necessary to figure out the influence of ball recovery patterns on team performance, such as the influence of different types of ball recovery on the attack success, and whether the attack efficiency is related to the zone where the ball possession is obtained, so as to be able to implement adequate training regimes and get objective feedback.

#### Running performance

Running performance was one of the most popular physical indicators in football matches [16,28,43], but increasing studies have found that running distance with possession of ball can distinguish the team’s match performance better, and it is a comprehensive indicator combining physical and technical variables [44].

## Discussion

The present systematic review aimed to investigate the interaction of the performance indicators related to ball possession with team performance. Through the in-depth analysis of the previous studies, it was decided that the most appropriate way to discuss the results would be the different presentation forms of ball possession, including ball possession percentage, ball possession strategy, duration, ball recovery patterns and running performance.

### Ball possession percentage

Ball possession has always been regarded as an important technical indicator to measure the match performance of a team, and it has also been widely studied in recent years [6]. The studies related to ball possession percentage mainly focus on the following aspects: the influence of ball possession percentage on the match success, the indicators that affect the status of ball possession percentage and the impact of different status of ball possession percentage on team performance, as presented in Table 1.

**Table 1:** The studies of ball possession percentage.

Authors	Sample	Purpose	Variables	Main results	Quality score(%)
Collet [38]	6078 matches from 2007–2010 seasons in EPL, Italian Serie A, French Ligue 1, German Bundesliga, La Liga, UEFA Champion League.	To explore the relationship between ball possession and the match success.	Goals, shots, league ranks and points, match venue, quality of teams, match outcome, pass to shots on goals ratio, passes, successful passes, ball possession.	The ball possession plays a positive role in match success, but its effect is not as significant as the shooting accuracy, especially in the close matches.	98.7
Lago-Peñas and Lago-Ballesteros [10]	380 matches during the 2008–2009 season in the Spanish Football League.	To investigate the influence of match location and the quality of opponent on the match performance in elite football matches.	Ball possession, goals, shots, shots on target, crosses, corners, loss of possession, fouls, passes, successful passes, foul committed, red and yellow cards.	Ball possession was significantly affected by match location and quality of opponents, home team had higher ball possession, away teams presented higher loss of ball possession.	98.2
Aquino et al [45]	380 matches played by 20 teams of the EPL 2015–2016 season.	To identify the effect of match location, match status, quality of opponent on the ball possession.	Ball possession, match location, quality of opponent, match status.	Home teams presented higher ball possession value than away teams. The possession was higher when play against weak teams than strong teams.	98.2
Kempe et al [46]	676 official games from 2009–2010 (n = 306) Bundesliga season, and the 2010 FIFA World Cup(n = 306).	To introduce two indicators to better characterize the playing styles in elite soccer match.	Passes per action, passing direction, target player passes, passing success rate, forward pass success rate, mean passes, gain of possession, ball possession rate.	Ball possession is related to the success of the match, but the most importance factor are control and effectiveness of attacking actions.	95.7
Lago-Peñas et al [7]	380 matches from the Spanish soccer league (2008–2009)	To identify which game-related statistics allow to discriminate winning, drawing and losing teams.	Total shots, shots on goal, play effectiveness,assists, crosses, offside committed and received, corners,ball possession,crosses against,fouls committed and against, and venue.	Total shots, shots on goal, crosses, crosses against, ball possession and venue were the variables that discriminate winning, drawing, and losing teams.	92.9
Aquino et al [17]	61 matches(988 player observations) played in the 2018 World Cup tournament.	To identify the variations of ball possession, match running performance, team network properties based on the match outcome and playing formation.	Ball possession, total distance covered, total distance covered in possession, total distance covered when out of possession, match outcome, playing formation.	Ball possession did not affect the match outcome. 4231 formation had a higher ball possession percentage than 442 formation.	92.6
Mota et al [29]	56 matches performed by 346 players in the 2014 FIFA World Cup.	To examine the effect of HPBPT and LPBPT on physical and technical indicators.	Distance at low, medium, high speed, sprints, passes, pass received, pass success, fouls, tackles, clearances, pass distance.	HPBPT or LPBPT does not affect the activity patterns of soccer match.	92.6
Bradley et al [16]	54 matches performed by 810 player from2013-2014 seasons in EPL	To examine the influence of situational variables on ball possession in EPL. To identify the variables that discriminate between HPBPT and LPBPT across different positions	Dribbles, shots, corners, goals, crosses, free kicks, successful and unsuccessful passes, fouls, fouled, events of tackles, tackled, clearance, and interceptions.	The ball possession was increased when losing than winning and drawing. The variables that discriminated between HPBPT and LPBPT were different for various playing positions, the successful passes was the most common discriminating variable.	89.6
Bradley et al [47]	54 matches performed by 810 professional players from 2013–2014 seasons in EPL.	To examine the effect of HPBPT and LPBPT on physical and technical variables in elite soccer matches.	Passes, successful passes, pass receives, touches per possession, dribbles, shots, goals, clearances, final third entries, possession won and lost, events of tackles and tackled.	HPBPT performed 44% more passes than the players in LPBPT. Total passes and passes received in HPBPT was higher across all playing positions than LPBPT.	89.6
Parziale et al [11]	123 matches from 2010–2011 seasons in EPL.	To determine the relationship between ball possession and winning.	Line types, ball possession rate, distance of passing, probability of ball retention.	Ball possession rate is strongly correlated with points earned during the EPL regular season.	89.4
Lago-Peñas and Martin [48]	170 matches during the 2003–2004 season in the Spanish Football League.	To identify the determinants of ball possession in elite football matches.	Ball possession, match location, match outcome.	The teams have more possession when they are losing than winning and drawing, the home teams have higher percentage of ball possession than away teams.	86.4
Dizdar et al [49]	88 matches from 2014–2015 season in the first Croatian football league.	To examine the influence of match location on ball possession.	The match location, match outcome, ball possession of host teams and guest teams.	The location of the match has no significant effect on the ball possession.	86.2
Goral [8]	56 matches performed by 32 teams from the 2014 FIFA World Cup	To identify the ball possession and passing success percentage of successful teams.	Ball possession percentages, pass attempted, passing success percentage, successful and unsuccessful passes.	Higher Ball possession percentage, especially in midfield and offensive third, successful passes, passing accuracy can significantly affect the match success.	86.2
Kubayi and Toriola [50]	32 matches played in PSL (South Africa Soccer League)2016-2017 season.	To examine the effect of situational variables on ball possession.	Match outcome, match location, quality of opponent, percentage of ball possession, duration of ball possession.	The losing teams had a higher ball possession percentage than winning teams. Home teams had higher percentage of ball possession than away team.	83.2
Shafizadeh et al [51]	32 matches from the 2006 FIFA World Cup and 23 national teams in the 2010 World Cup.	To identify the determinants of losing ball possession in the elite soccer matches.	illegal movement, clearance, ball control, delay, passing to the marked player, ball manipulation, teamwork.	The factors contribute to losing ball possession originated from motor and cognitive components.	80.1
Kubayi and Toriola [52]	30 matches performed by European (n = 15), African (n = 15) in the stage group of 2018 World Cup	To differentiate the key performance variables from European and African teams.	Ball possession, total passes, long passes, crosses, total shots, goals, shots on target,dribbles, corners, ball recoveries, tackles, yellow and red cards.	Ball possession was one of the most important indicators that differentiate European and African teams.	79.5
Tschopp and Cavin [53]	36 matches from 24 teams during the UEFA Euro 2016.	To evaluate how ball possession interacts with physical and technical indicators.	Total distance, high speed distance, goals, passes, successful passes, attempts, attempts on target.	Ball possession has no influence on game outcome. HPBPT teams performed more passes, successful passes, it only shows that HPBPT teams has a higher technical level performance.	78.9
Mohd et al [54]	12 matches performed between JDT and their opponents in the 2015 Malaysia Super League.	To analyze the relationship between ball possession and game outcome	Successful and unsuccessful passes, ball possession, shots goals, shots on target.	High ball possession will not significantly affect the outcome of the game, but it will increase the total shots of the team.	76.8
Merlin et al [55]	827 ball possessions from 6 matches in the 2016 Brazilian Championship League.	To find the technical variables that can most discriminate the ball possession.	The time of ball possession, passes, shots, goals, the length width, distance and effective playing space of both teams.	The passing, distance and width of receiving are the most important indicators that can affect the ball possession of the team.	76.8
Ajibua and Igbokwe [13]	6 matches played by Nigeria Female National Team in 2010 FIFA U-20 Female World Cup	To examine whether ball possession is a determinant factor of victory in female soccer.	Frequency of ball interception and ball control, throw-ins, free kicks, corners, goals, attempts at goal.	Ball possession is not the determinant of success in soccer games.	76.4
Clemente et al [37]	9218 instants from 6 matches in elite football.	To identify the ball possession status on the spatio-temporal relationship of players.	The area of play, weighted centroid, weighted stretch index, surface area.	The status of ball possession significantly affects players’ spatio-temporal relationships.	74.8

In the research on the relationship between the ball possession percentage and match success. Previous studies found that the ball possession percentage can significantly affect the outcome of the match, and the success of the game is often won by the team with a higher percentage of ball possession [7,11]. Goral and Kemal [8] confirmed the viewpoint in the study of the 2014 FIFA World Cup tournament, the lead in ball possession percentage is one of the important characteristics of successful teams, and winning teams often have a higher ball possession percentage, especially in the final third. However, Kempe et al. [46] regarded that the ball possession percentage may have a positive impact on the outcome of the game, but it cannot fully reflect the real situation of the game. A higher ball possession percentage does not mean that the number of shots and goals are higher, the key to match success lies in the quality of shooting [13] and the effectiveness of attack [38]. Conversely, the results of some studies [17,53,54] showed that the differences in the impact of ball possession percentage on the match outcome. For instance, Aquino et al. [17] reported that the ball possession percentage is irrelevant to the outcome of the game, but the match outcome is related to the playing formation of the team. Mohd et al. [54] reported that during the 2010 FIFA World Cup and the 2009–2010 Bundesliga season, there was no obvious relationship between ball possession percentage and the match outcome, but the match success was significantly influenced by the number of shots.

In the research related to the indicators that affect the status of ball possession percentage, previous studies have found that the factors include: match location [49], the quality of opponents [50], match status [47], playing formation [17], passes accuracy [8], cognitive ability and awareness of players [51]. For example, Kubayi and Toriola [50] reported that match location has significant effects on ball possession, the home teams had a higher ball possession percentage than the away teams. Which was in line with the findings from Aquino et al. [45]. However, Dizdar et al. [49] found that the home advantage cannot be represented in the ball possession percentage in the First Croatian Football League. Bradley et al. [47] and Maneiro et al. [19] demonstrated that the ball possession percentage was higher when the team was losing rather than winning. The reason for the increase in ball possession may be the desire of the losing team to regain the score. They are inclining to take more risky offensive actions to possess the ball and create the scoring opportunities, while the leading team focused their attention on the defense and consolidated its advantages. Aquino et al. [17] indicated that during the 2018 FIFA World Cup, the ball possession percentage of the team with 4231 formation had a higher ball possession percentage than 442 formation. The reason is that the 4231 formation team has wider passing routes and more diversified playing styles. Aquino et al. [45] reported that during the 2015–2016 season in EPL, home teams presented higher ball possession value than away teams, the ball possession percentage was higher when playing against weak teams than strong teams.

As for the studies on the impact of different status of ball possession percentage on team performance, Bradley et al. [16] analyzed the 2013–2014 seasons in EPL (English Premier League) and found that the total passes and passes received in HPBPT (high-percentage ball possession teams) was higher across all playing positions than LPBPT (low-percentage ball possession teams), and HPBPT performed 44% more passes than the players in LPBPT. Bradley et al. [47] reported that the variables that discriminated between HPBPT and LPBPT were different for various playing positions, the successful passes were the most common discriminating variable. Tschopp and Cavin [53] indicated that during the 2016 European Football Championship, HPBPT teams performed more passes and successful passes than LPBPT. But Mota et al. [29] indicated that during the 2014 FIFA World Cup tournament, the differences between HPBPT and LPBPT in technical and physical performance were not significant.

### Ball possession strategy

Team performance involves the interactions of technical, tactical and physical activities among players [56]. These interactions are the result of coaches’ tactical arrangement and game dynamics, and they can be explained by measuring the offensive and defensive behaviors of players and opponents of a team [57]. Previous studies have made some findings as shown in Table 2. Fernandez-Navarro et al. [58] found that possession play and direct play were the most commonly used tactical strategies during the 2006–2007 and 2010–2011 seasons in EPL and Spanish first division teams (La Liga). Lago-Peñas et al. [24] reported that the playing style of the Chinese Soccer League was various, including counterattack, direct attack, and possession play. Gonzalez-Rodenas et al. [59] reported that during the 2010 FIFA World Cup, the teams with possession type of play achieved more scoring opportunities than counterattack teams, and the scoring efficiency of set-piece tactics was also higher than ball recoveries and restarts. The value of ball possession percentage in the score box of counterattack teams was lower than the style of possession play teams, this may be due to the reason that counterattack teams were more inclined to organize attacks by long passes [60]. Yi et al. [61] recorded that during the 2018 FIFA World Cup, the passing, goal scoring performance of possession play teams was superior than direct play teams. But Sarmento et al. [26] indicated that the offense efficiency of counterattacks is 40% higher than positional attacks. Gonzalez-Rodenas et al. [59] demonstrated that counterattack was more efficient than mixed play and direct attack only when the opponent’s defense was weak, and the long passing sequence could achieve more scoring opportunities when the defense was organized.

**Table 2:** Studies of the ball possession strategies.

Authors	Sample	Purpose	Variables	Main results	Quality score(%)
Gómez et al [62]	301 matches from 2013–2014 seasons in the Greek Superleague football teams.	To investigate the playing style of Greek Super league football teams according to match location, the quality of opponents.	A total of 62 variables were analysed, including 40 variables related to attacking, 8 variables related to defending, and 6 variables related to transition 8 variables related to set pieces.	Home teams perform superior in ball possession, ending actions, set-piece, transition-play, fouling actions than away teams. The high ranking teams obtain greater value in ball possession and ending actions, the low-ranking teams obtain higher value in individual actions.	98.8
Castellano and Pic [22]	373 matches performed by 20 teams from 2016–2017 seasons in Spanish La Liga.	To identify the playing style of teams according to the technical performance.	Ball possession percentage, ball possession in different areas, the percentage of counterattacks in total attacks, passes, dribbles, successful passes, total distance covered.	Teams should have the ability to execute various tactics, and would better to formulate playing styles around the technical characteristics of key players.	98.4
Lago-Peñas and Dellal [12]	380 matches from 2008–2009 seasons in EPL	To examine the effects of situational variables on ball possession strategies.	Multiple-camera match analysis system was used. Ball possession, match location, match status, and the quality of the opponents was analyzed.	Possession strategy was influenced by situational variables. Team possession percentage was higher when losing than winning and losing. Home teams have greater possession than visited team. The strong opponent was associated with a reduction in time spent in possession.	97.2
Gollan et al [63]	380 matches in the 2015–2016 season from Spanish LaLiga.	To evaluate the influence of contextual factors on the playing styles in elite matches.	Match venue, the quality of opponents, goals, playing styles.	Home teams were more likely to counterattack or offensive attack when against the defensive teams; Teams were more inclined to counterattack when against Top 10 teams; Teams were more likely to play offensive attack when against Bottom 10 teams.	97.2
Fernandez-Navarro [64]	380 matches of the English Premier League from the 2015–2016 season.	To identify the effect of match status, match location, the quality of opponents on the style of play in football matches.	Situational variables, the playing effectiveness of direct play, counterattack, maintenance, build up, sustained threat, fast tempo, crossing and high pressure.	The match status have a significant effect on all styles of play; the match location have a positive impact to all playing style except counterattack and maintenance; the quality of opponents have a significant effect on all styles of play except counterattack.	94.6
Sarmento et al [26]	68 matches from LaLiga, Italian Serie A, German Bundesliga, English Premier League and Champions League.	To identify the influence of tactical and situational variables on offensive sequences in elite football matches.	Type of attack, the beginning of the offensive process, the end of the offensive process, spatial area of field, interactional context in the center of the game.	The playing style of counterattack increased the success of an offensive sequence by 40% compared with positional attacks. The attacks originated in pre-offensive or offensive zones were more successful than those started in the defensive zones.	94.1
Fernandez-Navarro [58]	97 matches from 2006–2007 and 2010–2011 seasons in EPL and La Liga.	To define the different playing tactics in elite football.	The ball possession in defensive, middle, attacking, central and wide area, the number of passes in different directions and areas, crosses and shots, the ball regains in different areas.	Direct play and possession style of play are the most general style.	93.6
Hughes and Franks [5]	116 matches performed by 56 teams during the 1990 and 1994 FIFA world cup.	To compare the differences of technical performance in successful and unsuccessful teams	Ball possession, total and per shots, goals, passes, passing sequence,	The ratio of shots to goals is higher for direct style of play than possession play.	92.1
Yi et al [61]	59 matches inthe 2018 FIFA World Cup	To examine the effect of different playing styles on the match performance.	Ball possession, variables related to goal scoring, attacking and passing, defending, physical variables including clearance, foul, total distance, sprint, top speed.	Possession-play characterised teams performed better in goal scoring, passing and attacking performance.	91.2
Lago-Peñas et al [24]	240 matches from 2016–2017 seasons in Chinese Soccer League.	To identify the different playing styles in professional football teams.	The type of play, ball possession, ball possession in offense half, counterattacks, elaborate attacks, passes, successful passes, set pieces, goals, shots, interceptions, tackles.	The playing style of each team are diverse,including counterattack, transitional play, and possession tactics.	90.8
Tenga et al [25]	163 matches performed during the 2004 season in the in Norwegian football league.	To investigate the effect of different playing styles on the the ball possession in score-box.	Ball possession, possession type, possession outcome, initiative zone, pass penetration, passes, pass length, defensive pressure, backup and cover.	The type of counterattack was more effective than elaborate attacks in unbalanced defence, the elaborate attacks was more effective in the balanced defence.	89.6
Harrop and Nevil [9]	46 matches performed in the 2013–2014 season from the Football League One.	To identify the technical indicators that discriminate the winning, drawing and losing of the teams.	Ball possession, passes, successful passes, dribbles, shots, shots on target, goals, style of play, match location, match outcome.	The successful teams performed more successful passes and shots, and more inclined to direct style of play.	85.8
Sarmento et al [36]	36 matches from during in 2009–2010 seasons.	To analyse the playing styles of elite football teams through their offensive organization.	The type of play, initial of attack process, tackle, interception, disarm, save, goals, shots, free kicks, corners, penalty kicks, passes, mistakes, fouls.	The style of play was based on the coach philosophy, technical and tactical levels of players.	83.5
Lago-Peñas [65]	27 matches from 2005–2006 seasons in La Liga.	To investigate the effect of match location, match outcome, and quality of opponent on the ball possession strategies.	Match location, match status, quality of opponent, ball possession, intercepts.	Possession strategies were significantly influenced by match location, match status, quality of opponents.	79.2
Lago-Ballesteros et al [23]	12 matches played in the 2009–2010 season from the Spanish soccer league.	To investigate the effect of different playing styles and situational variables on the the ball possession in score-box.	Possession type, duration, initiative zone, passes, defender number, defensive pressure, match location, match outcome, quality of opponent.	The type of counterattack and direct play were three times more effective than elaborate attacks in creating ball possession in score-box.	78.2
James et al [66]	21 matches played by 29 players during the 2001–2002 season in British and European matches.	To evaluate the possession strategies of a team in British and European matches.	Ball possession time, passes, assists, the contribution of players, the ratio of easy to difficult passes of players.	The attacks occurred more frequently down the right side zone in British competitions than European matches.	74.4
Gonzalez-Rodenas et al [59]	7 matches played in the World Cup 2010, 857 team possessions were analysed.	To examine the relationship among the playing styles, situational variables and scoring opportunities.	The type of team possession, initial zone, the number of initial defenders, passes, duration, pass success, match status, match venue, score line, possession outcome.	The scoring opportunities by set plays are greater than recoveries and restarts, more scoring opportunities were achieved in offensive zone, initial penetrative actions, low number of initial defender, the playing style of counterattack, longer passing sequences and duration.	73.2

Lago-Peñas and Dellal [12] reported that during the 2008–2009 season in EPL, the ball possession strategy was influenced by situational variables, and the ball possession time would be reduced when the team was ahead. When the home team plays against the away teams with defensive tactics, they tend to adopt the ball possession strategies such as counterattack or aggressive tactics. Regardless of home advantages, when against strong teams, it is more likely to adopt a defensive playing style and counterattack than an elaborate attack. And the likelihood of adopting an aggressive playing style was higher than a defensive playing style and counterattack when against weak opponents. This is in accordance with the results of several studies [7,25,48,64]. Sarmento et al. [36] compared the ball possession strategies for the two consecutive seasons from 2013 to 2015 in EPL, La Liga, Serie A and Bundesliga League and pointed out that the playing styles in different leagues were influenced by many factors, such as regional culture, coaching philosophy [22] and the technical and tactical ability of players [65]. English Premier League teams prefer direct attack. Serie A teams are more inclined to traditional defensive style, while La Liga teams advocate the tactical style of ball possession. However, the direct attack of EPL, which is characterized by long passing, can make the penetrating attack in the final third more effective, and the number of threatening attack opportunities per game is three times that in the European Champions League, although it is closely related to the disparity of opponents in the domestic league. And Castellano and Pic [22] indicated that the ball possession strategies of the teams should be flexible to formulate tactics according to the situation on the field and around the key players.

### Ball possession duration

The duration of each ball possession is the epitome of the team’s tactics [25]. Reep, Benjamin [67], Hughes and Franks [5] reported that the shorter the ball possession time and short passing sequence could create more opportunities for goal scoring. As Table 3 presented, Harrop et al. [9] found that 38.8% of the possession lasted for 5–12 seconds, while the possession lasted for more than 12 seconds accounted for 37.3% in La Liga. This shows that the teams in the English Premier League prefer the tactics of direct attack, while the teams in La Liga prefer the patience of passing and organizing. Moreover, the players in La Liga usually have prowess individual ability and brilliant passing and receiving skills, which is one of the reasons why they can maintain a high ball possession percentage. At the same time, the La Liga team scored more goals in the ball possession duration for more than 12 seconds than the EPL teams, which also reflects that the La Liga teams were not only higher than the EPL teams in the frequency of passing, but also higher in the success rate of shooting. In addition, Lago-Ballesteros et al. [23] found in the La Liga in the 2009–2010 season that the La Liga teams had longer possession time than EPL teams in the penalty area. This trend is consistent with the findings of Tenga et al. [42], who discovered the most goals were scored when the possession time lasted for more than 12 seconds, and the number of possessions lasted more than 12 seconds was significantly larger than the bottom teams.

**Table 3:** Studies of the duration of ball possession.

Authors	Sample	Purpose	Variables	Main results	Quality score(%)
Dellal et al [27]	380 matches performed by 3540 players from 2005–2006 season in the French First League.	To identify the physical and technical performance according to different playing positions in elite soccer matches.	The total distance covered in high-intensity, sprinting, the success of ground and aerial duels, successful passes, ball possession duration, ball touches per individual possession.	The ball possession duration of the players were between 55.5s and 74.2s per match, and they had less 2.2 ball touches per individual possession.	96.7
Mota et al [29]	55 matches performed by 346 players from the 2014 FIFA World Cup tournament	To examine the effect of HPBPT and LPBPT on physical and technical variables during 2014 FIFA World Cup matches.	Total running distance, high-intensity running distance, passes, passes received, pass success,tackles, fouls and clearances.	Ball possession does not influence the activity patterns of international matches although HPBPT spend more time in offensive areas of the pitch.	92.8
Link and Hoernig [68]	60 matches in the 2013–2014 German Bundesliga season	To describe individual and team ball possession models according to different playing position in elite soccer matches.	Individual ball position, ball action, ball control, team ball possession, team ball control, team playmarking.	Central forwarders has the shortest individual ball control times 0:49 ±0:43min, the longest for goalkeeper 1:38±0:58 min, central defenders 1:38±1:09 min and midfielders 1:27±1:08 min.	92.6
Maneiro et al [19]	3740 ball possessions from 52 matches performed in 2015 Women’s World Cup.	To investigate the influence of variables on the ball possession.	Half time, initial zone of attack, the type of play, possession zone, passes, goals, shots, score line, match outcome.	There are significant differences between successful and unsuccessful teams based on match status. Unsuccessful teams had longer possession time when losing rather winning.	92.4
Tenga and Sigmundstad [42]	997 goals in the Norweigan top league (2008–2010) season.	To compare the type of ball possession in the open play between teams from top, in-between and bottom teams from professional soccer league.	Possession type, the passes of per possession, possession duration and possession starting zone.	The top three teams on average scored significantly more goals started in the midfield zone than bottom three teams. The possessions duration >12s, and the attacks initiate in central zone was lower than top three teams.	89.6
Jones et al [69]	24 matches performed by successful and unsuccessful teams in 2001–2002 EPL season.	To identify the differences in duration of possession between successful and unsuccessful teams.	Ball possession, duration of possession(3-10s, 10-20s, 20s+), match outcome.	Both successful and unsuccessful teams hold longer duration of possession when losing compared to winning.	78.2
Casal et al [18]	12 matches in the knockout stage of the 2016 UEFA Euro tournament.	To determine whether possession time and field zone of possession are performance variables to distinguish the successful and unsuccessful teams.	Possession time, possession zone, match outcome, match status, match half, move outcome	The successful teams had longer possession times, prefer in the middle offensive zone. Unsuccessful teams had shorter possession times, and prefer in the middle defensive zone.	77.5
Gonçalves et al [41]	12 matches played from 2018–2019 season in EPL.	To determined the spatial and temporal characteristics of teams according to the quality of opponents.	Ball possession duration, ball position, the initial, final zone of ball possession, game length, game width, the quality of opponents.	The possession duration was significantly affected by the opponent’s quality. When against strong opponents, the average ball possession duration lasted for 28s, and 37s for weak opponents.	76.8
Andrzejewski et al [70]	147 players participating in 10 matches of the 2008–2009 and 2010-2011European League season	To analyze the physical and technical activities of elite soccer matches.	Total running distance, sprint distance, the number of ball possessions, ball touches per possession, duration of ball possession, successful passes.	Players retained ball possession between 36.4s and 64.9s per match,they had no more than 2.3 ball touches per individual possession.	74.2

However, the findings differed from the diverse seasons and competitive level matches. Tenga et al. [42] found the number of goals that scored in less than 5 seconds attacks in EPL was higher than La Liga teams, which demonstrated that EPL teams had higher attacking efficiency in counterattack. In the 5–12 seconds possessions, the probability of scoring goals in La Liga is 0.3%, which is lower than that in Premier League (0.7%), but the number and efficiency of attacks of La Liga were higher than EPL teams in >12 seconds possessions. Sarmento et al. [26] reported that during the 2013–2015 two consecutive seasons in La Liga, Premier League, Serie A, and Bundesliga, the possibility of creating a successful attack opportunity will decrease by 2% when the possession duration of the team increases by one second, and the possibility of creating a successful attack opportunity will decrease by 7% when the number of passes increases. When considering the influence of situational variables on the possession time, Bradley et al. [47] investigated the matches in the 2013–2014 seasons in EPL and found that the number and duration of possessions could increase when against weak teams, and when the teams were losing, the team will pay more attention to fighting for the ball possession in the final third, they will not keep the ball too long in the defensive half, hence, the possession time in attacking half will increase, and the more rank points a team had, the longer duration time in ball possession [16]. But Taylor et al. [71] found that the quality of opponents will not affect the team’s overall technical performance.which is lower than that in Premier League (0.7%), but the number and efficiency of attacks of La Liga were higher than EPL teams in >12 seconds possessions. Sarmento et al. [26] reported that during the 2013–2015 two consecutive seasons in La Liga, Premier League, Serie A, and Bundesliga, the possibility of creating a successful attack opportunity will decrease by 2% when the possession duration of the team increases by one second, and the possibility of creating a successful attack opportunity will decrease by 7% when the number of passes increases. When considering the influence of situational variables on the possession time, Bradley et al. [47] investigated the matches in the 2013–2014 seasons in EPL and found that the number and duration of possessions could increase when against weak teams, and when the teams were losing, the team will pay more attention to fighting for the ball possession in the final third, they will not keep the ball too long in the defensive half, in hence, the possession time in attacking half will increase, and the more rank points a team had, the longer duration time in ball possession [16]. But Taylor et al. [71] found that the quality of opponents will not affect the team’s overall technical performance.

### The patterns of ball recovery

The team success of elite football teams also depends on the patterns of ball recovery, which includes the types of ball recovery and the zone that the ball is regained. As for the types of ball recoveries, previous studies have not reached a consensus in this respect (as shown in Table 4). Oberstone et al. [72] found that the most common ball recovery type in EPL was an interception. While in La Liga, set-plays (29.2%) were the most common ball recovery type in La Liga. The reason for this difference may be that the penalty scale of referees in EPL was not as strict as that in La Liga, and the number of set pieces was less. Barreira et al. [30] investigated the ball recovery patterns in the different zones on the pitch during the 2010 World Cup tournament and found that the tackles usually occurred in the defend third, and the interception mainly occurred in the central zone. In summary, the frequency of direct ball recovery type (interceptions, tackles, goalkeeper saves) was higher than indirect ball recovery type (set pieces, turnover). Rowlinson and Donoghue [73] found during the knockout stage of 2012 UEFA Championship League matches, the tackle was the most common type of ball recovery. Several findings were consistent with this viewpoint [74–76]. The reason for the differences in the type of ball recoveries may be due to the situational variable effects. When the team was losing, the defensive pressure of the winning teams was increasing, and the probability of making mistakes of defenders would increase as well. It was more likely to seem that the defenders kicked the ball out of the pitch for clearance. In that way, the set pieces may also become the most common ball recovery type.

**Table 4:** Studies of the patterns of ball recovery.

Authors	Sample	Purpose	Variables	Main results	Quality score(%)
Vogelbein et al [32]	306 matches of 2010–2011 German Bundesliga season.	To examine the time of ball recovery that the teams required, identify the differences and influences between German Bundesliga football teams.	The aerial challenges, tackles, clearances and interceptions, the time of ball possession recovery, the time of losing ball possession.	The time of ball regain was a critical factor of defensive performance in elite football teams. The faster the ball was regained, the better the defensive performance of the team will be.	95.8
Jamil [31]	106 matches from EPL season (2015–2016 and 2017–2018)	To examine possession regains patterns of elite soccer matches.	Possession regains, clearance, quality of opponent, venue, short passes, corner success rate, successful crosses.	Players regain the ball possession on the left side was more productive than the central zone or right side.	91.2
Hughes and Lovell [77]	3077 transitions from the 29 matches in the 2014–2015 UEFA Championship League.	To analyse the characteristics of transition in the elite soccer matches.	The type of ball recovery, ball possession, passes, dribbles, successful passes, shots, shots on target, goals, possession outcome, match outcome.	The tackle was the most effective type to create scoring opportunities. Successful teams created more scoring chance from the defensive half, but more goal chances was produced from the offense area when losing.	86.8
Barreira et al [30]	1619 attacks performed by the 2010 FIFA World Cup semi-finalists (Germany, The Netherlands, Spain and Uruguay)	To characterize ball possession recovery patterns in related to pitch zones, competition stage and teams, and to analyse the relationship between the ball possession recovery and attacking events.	Type of ball possession recovery, attacking play efficacy, match status, competition stage, match time, duration of the attack and any match events.	Direct ball possession recovery was higher than indirectly recovery. The ball was most often regained in defensive and mid-defensive central zones, throw-in was the only type of ball possession recovery that differentiated semi-finalists. The ball possession recovery in mid-defensive central zones increasing the attacking efficacy.	84.5
Almeidaet al [78]	28 matches performed by 16 elite teams during the 2011–2012 UEFA Championship League.	To examine the effect of match location, quality of opponent and match status on the ball recovery patterns.	Match location, match status, opponent’s quality, tackle, interception, turnover, set play and save, ball recovery zone:defensive, offensive, offensive, defensive midfield zone.	Interceptions regain more possessions than other type. Most ball possessions were regained in defensive zone. Match status, match location and quality of opponent have interactive effects on the defensive performance.	84.2
Shafizadeh et al [79]	32 national teams attended 2010 World Cup, and 12 soccer clubs from the 2012–13 NexGen Cup and the EPL	To examine the temporal occurrence of losing possession of the ball in soccer and its association with conceding a goal.	Transition time, time of losing the ball, the reason of losing ball, the time of goal conceding, the time interval between losing possession.	The number of ball losts increased the goals conceding, the longer duration of ball lost was, the more likely to goal-conceding.	84.2
Fernandes et al [57]	28 matches of the four semi-finalist teams of the 2014 World Cup	To examine the ball recovery patterns of successful teams and its influence to tactical modelling, halves, match status, opponent quality and stage competition.	Match status, opponent quality, halve, type of stage of competition, type of development, type of subphase, defenders tactical and technical actions.	Germany were more inclining to perform ball recovery by the goalkeeper than Argentina or the Netherlands.Team facing lower-ranked opponents were less likely to perform ball recovery by interception.	82.6
Barreira et al [74]	24 matches played by the semi-finalist in the 2010 World Cup tournament.	To characterize ball recovery patterns and investigate the influence of each type of ball recovery on the subsequent patterns of attacking play.	The ball recoveries in left, right, midfield offensive and defensive zone, the patterns of attack play.	The offensive, defensive zone do not seem to be significantly associated with ball recovery behaviour.	82.6
Sgrò et al [60]	31 matches played in the last stage of the 2012 European football Championship.	To examine the effect of situational variables on the probability of achieving score-box possession.	Possession type, passes, starting paths, starting zones, quarters, match status, halves, quality of opponents, level of tournament.	The offense started in the right path was worse than the left path, the stronger the opponent was, the lower the ball possession percentage was.	80.2
Cooper and Pulling [40]	20 matches from the 2017–2018 season of EPL and La Liga.	To investigate the impact of the ball recovery type, location of ball recovery, and the duration of the possession on the outcomes of possessions.	The location of ball recovery, the type of ball recovery (interception, tackle, goalkeeper save, set-play and turnover) and the duration of possession	EPL teams performed more goals and shots when recovering possession through turnovers, La Liga teams scored relatively more goals after tackles.	79.8
Casal et al [80]	804 defensive transitions fromeight matches (quarter-finals, semi-finals, and finals)of the 2010 World Cup	To identify variables associated with the direct recovery of ball possession.	Possession loss zone, duration of defensive transition, defensive organization, zone of offensive and defensive transition.	The variable that most strongly associated with recovery of the possession of the ball is the area in which the ball is lost, offensive transition accomplished within 15s increased the likelihood of directly ball recovery.	79.2
Taylor et al [81]	22 matches during the 2003–2004 season in the professional British football league.	To evaluate the possession strategies, technical and tactical performance in elite football matches.	Ball possession, passes, shots, goals, clearance, foul, offside, dribbles, crosses, duels, interceptions, tackles, losses of control, start area.	There is a significant differences in offensive efficiency among the teams on the right side, and the weak area in the defensive area of each team is on the left side.	78.8
Maleki et al [82]	28 matches of semi-final teams in the 2014 World Cup tournament.	To investigate the ball recovery performance in the match within six time periods.	Tackles, interceptions,saves,set play, turnover won across four field zones(offensive, mid-offensive, defensive, mid-defensive,)	Most ball recoveries were performed in the defensive and middle-defensive zones.	78.4
Casal et al [18]	2284 attacks from 12 matches in the knockout stage of the 2016 UEFA Euro tournament.	To identify the difference in possession area between successful and unsuccessful teams.	Possession time, possession zone, match outcome, match status, match half, move outcome	Successful teams have significantly longer possession time in middle offensive area, and the ball possessions in central zone significantly affects the offensive efficiency of teams.	76.8
Santos et al [83]	608 offensive sequences in 7 matches performed by Spanish Team in the 2010 World Cup tournament.	To verify patterns of ball recovery.	The number of ball recovery in right, midfield, left zone, offensive, defensive zone.	The Spanish Team performed more ball recovery in the right defensive midfield zone, the central offensive zone provided less recovered balls.	73.9

Although most ball possessions were regained by interceptions, it was difficult to convert the interceptions into goals [72]. Hughes and Lovell [77] found that 0.82% of interceptions resulted in goals and 12.12% of interceptions completed shots during the 2014–2015 UEFA Championship League tournament, which is much higher than the shooting accuracy after an interception in the English Premier League (5.7%) and La Liga (7%). This coincided with the viewpoint of Wright et al. [84], who discovered that only a low proportion of shots or goals were preceded by interceptions. However, there are also some studies considering that the attack efficiency was higher when the ball possession was regained by tackles. Cooper and Pulling [40] found that during the 2017–2018 season in the EPL and La Liga, the team from La Liga had superior scoring efficiency (2.5%) compared with the team from the EPL after tackling. Hugh and Franks [5] reported the most goals were scored in the possession regained by tackles in La Liga. This is because the teams in La Liga can better grasp the opportunities of unbalanced defense, properly dominate the tempo of attack after tackling, and can take advantage of the chance to launch a threatening attack.

In the studies of the effects on the ball recovery types, the area of losing ball possession was most related to the type of ball recovery [80], but Barreira et al. [30] disapproved of the view by studying the matches in 2010 FIFA World Cup, who reported that the zone of ball recovery was not significantly associated with ball recovery actions. Alemida et al. [78] discovered that the match location could affect the type of ball recoveries, the home teams performed better defensive performance, so the possession regained by goalkeeper saves was the least. In the matches between teams were at a similar level, the quantities of ball possessions regained by tackle were higher than the unbalanced matches, the differences may be influenced by the tactical strategy and mistakes that players made [57]. For example, in order to maintain a high ball possession percentage to dominate the match tempo, they needn’t take much defensive action against weak teams for regaining ball possession. In addition, the scoreline will also affect the type of ball recovery [32]. When the team was losing, they will duel more fiercely to regain ball possession and organize the attack, while the leading team will focus on the defense task and will not take too aggressive defensive actions.

The area where the ball possession was regained marked the initiative zone of offense [83]. Tenga et al. [42] recorded the Norweigan top league for three consecutive seasons from 2008 to 2010 and found that most of the ball possession exchanges took place in the defensive half. Makleki et al. [82] supported these findings by studying the elite matches in the 2014 FIFA World Cup and found that most ball recoveries were performed in the defensive and middle-defensive zones. Barreira et al. [74] observed that during the 2010 FIFA world cup, the ball possession recoveries often occupied in the defensive third, but the champion of the tournament regained most ball possessions at the right side, the least at the central zone. Jamil [31] also found this tendency when observing the EPL matches and complemented that this phenomenon can be explained from the tactical point of view. The tactical play of the Premier League teams prefers carrying out high-intensity defense in the final third, which leads to the midfielder of opponents being can not easily organize the offense and only releasing the ball to both sides. Therefore, the quantities of ball possession regained in the attacking half were larger than the defensive half, especially in the side area.

In the comparative study of attacking efficiency of regaining the ball possession in different areas, Tenga et al. [42] declared that the attacking efficiency of the successful teams in different zones of pitches was higher than unsuccessful teams, especially when the ball recovery occurred in the defensive zone. Since most successful attacks initiated in the defensive zone require players to achieve penetration with well-timed runs and accurate passes at a high tempo, which is often rarely performed by the players of unsuccessful teams. Hughes and Lovell [77] observed the knockout stage of the Champions League in the 2014–2015 season and found that the goal scoring opportunities created by the ball regained in the offensive area were 7 times higher than the defensive area, and the number of goals scored was 11 times higher. Some studies have pointed out that the attacking efficiency of the ball regained in the offensive area was higher, such as the number of goals, shots, and set-piece won [59]. Specifically, Cooper and Pulling [40] found that most goals were achieved after the ball was regained in the attacking area, while the ball possession obtained from the defending area scored fewer goals. Casal et al. [18] found that most of the threatening attacks were launched after the ball was regained from the mid-defensive area and the mid-offensive area, and the attacks initiated from the mid-offensive area were even more threatening, because it was the weakest moment for the team to transition from attack to defense, and it was easy for opponents to goal scoring in the situation of imbalanced defense.

### Running performance

Running performance in football matches has been widely discussed by experts over the last two decades. As Table 5 depicted. The previous focused on the running distance at the different speed categories, but there are few studies on the association between running performance and ball possession [85].

**Table 5:** Studies of the running performance with possession of the ball.

Authors	Sample	Purpose	Variables	Main results	Quality Score(%)
Dellal et al [27]	600 matches performed over 2006–2007 seasons in EPL and Spanish La Liga.	To compare the technical and physical performance differences between the two national leagues.	The total distance covered, distance covered at high-intensity speed and sprint, the distance covered with or without ball possession.	La Liga teams covered more distance than EPL when in possession because of the difference in ball possession strategies.	98.2
Brito et al [43]	1520 matches performed by 20 elite teams across four consecutive seasons (2015–2016 and 2018–2019) in the Spanish La Liga.	To examine the association between running performance and with or without ball possession in the elite soccer matches.	The total distance, distance covered at different speed category, distance covered with or without ball possession.	The top ranked teams covered more distance with ball possession than middle, bottom ranked teams, the unsuccessful teams covered more total distance but the contribution is limited.	98.2
Rampinini et al [21]	416 matches performed by 186 players in Italian Serie A league.	To investigate the difference of physical and technical performance between first and second half in the elite soccer matches.	Total distance covered, running distance at high-intensity,very high-intensity, with or without ball possession, the number of skills, passes, successful passes, shots, goals, tackles, dribbles.	The more successful teams covered more total distance and high-intensity running distance in possession of the ball.	96.1
Gorki et al [86]	306 matches across 2012–2013 season in German Bundesliga.	To identify the association between running performance and match success in the elite soccer matches.	Total distance covered, the number of running at high-intensity, very high-intensity, sprinting, with or without ball possession, match outcome.	There is a positive correlation between the match success and the total running distance with ball possession.	94.2
Yang et al., [87]	240 matches of the Chinese Super League in the 2014–2015 season.	To investigate the physical and technical performance related to team quality.	Total distance, distance covered in sprint, high-intensity, high speed, total distance in and out of ball possession, distance covered out of ball possession in sprint, high-intensity, high speed.	High ranked teams have covered more total distance, sprint, high-intensity, high-speed distance with ball possession than lower ranked teams.	92.6
Aquino et al [17]	61 matches played by 988 players in the 2018 Russia FIFA World Cup tournament.	To examine the variations of running performance and ball possession based on the playing formation and match outcome.	Ball possession percentage, ball possession in defence, midfield and attack zone, total distance covered, total distance covered with or without ball possession, the distance covered in walking, moderate speed, high-speed, very high-speed, sprinting and maximum speed.	The match outcome was not significantly affected by the possession rate and running performance. The teams employing 4231 formation had higher ball possession and covered more distance with ball possession.	91.8
Mota et al [28]	55 matches performed by 346 players in the 2014 FIFA World Cup tournament.	To examine the influence of ball possession status on the running performance in the elite soccer matches.	The running distance covered by low, medium, high, very high, sprint speed, ball possession status, running distance with or without ball, effective play time.	The total running distance and the medium speed distance in HPBPT were lower than LPBPT, LPBPT covered more distance without ball possession, less distance with ball possession.	89.4
Bradley et al [16]	54 matches performed by 810 players in EPL.	To investigate the influence of HPBPT and LPBPT on the running performance in the elite soccer matches.	The total distance covered by standing, walking, jogging, running, high-speed, sprinting, the distance covered with or without ball possession.	The total distance had no significantly differences between HPBPT and LPBPT, but HPBPT had more 31% running distance (with ball possession) in high-intensity than LPBPT, but 22% less distance without ball possession.	88.2
Ade et al [44]	46 home matches performed by 20 players across four consecutive 2010–2011, 2013–2014 seasons in EPL.	To examine the association between movement patterns, technical and tactical performance according to different playing positions,	Movement patterns, match location, technical skills, tactical activities and combination play.	The mean running speed with ball of wide midfielders were faster than other positions. When out of possession, forwards made more efforts running than other positions.	86.9
Gregson et al [88]	57 matches performed by 485 players from 2003–2004 season in EPL.	To identify the variation of high-intensity running performance in the elite soccer matches.	Total high speed distance covered, total sprint distance covered, total distance with or without ball possession.	The high-intensity running variability was greater when the teams with the ball possession than they were out of ball possession.	85.4
Miguel [89]	8468 matches observation performed by 412 players in Spanish La Liga (2018–2019) season.	To investigate the association between the possession time and running performance.	Total distance covered, low, medium, high, very high, and sprint speed distance covered, effective playing time.	The running distance of VHPBPT was lowest, especially in low and medium speed, and attackers covered lowest distance. Backs in VLPBPT covered lowest running distance.	82.6
Caring [90]	30 matches player over 2007–2008 and 2008–2009 seasons in French League 1 division.	To identify the running performance with the ball in the elite soccer teams.	The distance covered in possession, distance covered in light speed, low speed, moderate speed, high speed and sprinting speed.	The average running distance with ball of player is 191±38 m, mean speed per possession is 12.9± 1.8 km·h^-1^, the peak speed with ball performed by wide midfielders and lowest is full-backs.	78.3
Bradley et al [91]	28 matches performed by 370 players from 2005–2006 season in EPL.	To investigate the high-intensity running performance according to different playing positions in the elite soccer matches.	The total distance covered in standing, walking, jogging, running, high-intensity running, sprinting, with or without ball possession, maximum speed.	The distance covered with and without ball in the last 15-min was greater than the first 15-min.	76.8
Mascio and Bradley [92]	20 matches played by 100 player from 2006–2007 season in English Premier League.	To investigate the running performance in high-intensity period during the elite soccer matches.	The total distance covered in standing, walking, jogging, running, high speed running, sprinting, with or without ball, maximum speed.	In the high-intensity period, players covered more distance in out of ball possession, but performed more 10% higher intensity running when in possession.	76.8

During the 2007–2009 two consecutive seasons in French League 1, the average running distance with the ball of possession was 191 ± 38 m, the mean speed per possession was 12.9 ± 1.8 km·h-1 [90]. Bradley et al. [16] found that there was a significant difference in the running distance with or without the ball. This finding coincided with that of Mota et al. [28]. Miguel [89] complemented that the total distance covered and the distance in out of ball possession for HPBPT was lower than the LPBPT, especially at the low and medium speed, but the distance covered with the ball was higher. Dellal et al. [20] made a comparative study of La Liga and the English Premier League in the 2006–2007 season and found that the La Liga teams covered more distance in the possession with the ball due to the differences in ball possession strategies. The La Liga teams attacks with long passes.

There are also some studies that the running distance with balls is related to other factors [21]. In particular, the top ranked teams performed more distance with the ball than the bottom teams. The unsuccessful teams covered more distance without the ball possession, but the contribution was limited [43]. Aquino et al. [17] examined the running performance in the 2018 FIFA World Cup and reported that 4231 formation covered more distance with ball, and the match outcome was not significantly affected by running distance with or without the ball of possession. Conversely, in the matches from 2012–2013 season in German Bundesliga, Hoppe et al. [93] found that the match outcome was related to the running distance with the ball, and the greater the running distance with the ball, the more likely the team will win. As for different playing positions, the wide midfield covered the greatest running distance with ball possession, while the forwards covered the greatest running distance without the ball [44]. In the different competition periods, Bradley et al. [91] observed the matches from 2005–2006 season in EPL and found that the distance covered with ball possession in the last 15-min was greater than the first 15-min, this may be due to the players’ willingness to reverse the score, which prompted them to cover more distances.

## Conclusion

The main purpose of the review was to explore the impact of variables related to ball possession on the match outcome and team performance from the following perspectives: ball possession percentage, possession strategy, ball possession duration, ball recovery patterns, and running performance with possession of the ball.

After reviewing all the included articles, the ball possession percentage is not dominant to predict the match success. The status of ball possession percentage can affect the team’s performance in passing, organizational and running distance with the ball possession. There are league differences in ball possession strategies and duration. For instance, English Premier League teams prefer the direct style of play, and the attack efficiency is higher in a short duration time. However, the main playing style of La Liga teams is elaborate attack, which can achieve more goals through long duration time. The frequency and offensive efficiency of direct ball recovery types are higher than indirect types. Ball possessions regained in the defensive third were higher than the final third. Finally, these variables related to ball possession are significantly affected by contextual variables, among which the main factors are match location, the quality of opponent, and match status. However, there remain some limitations such as the difference in the definition of concepts and sample participants, only a few studies consider the influence of situational variables, lack of in-depth analysis on ball possession strategy. Therefore, further study should adopt a more comprehensive approach, especially establishing a new connection between possession strategy and more technical, tactical and situational variables.

## Supporting information

S1 Checklist (DOC)
